# Potential Application of ^1^H NMR for Routine Serum Lipidome Analysis –Evaluation of Effects of Bariatric Surgery

**DOI:** 10.1038/s41598-017-15346-0

**Published:** 2017-11-14

**Authors:** Adriana Mika, Zbigniew Kaczynski, Piotr Stepnowski, Maciej Kaczor, Monika Proczko-Stepaniak, Lukasz Kaska, Tomasz Sledzinski

**Affiliations:** 1 Department of Environmental Analysis, Faculty of Chemistry, University of Gdansk, Wita Stwosza 63, 80-308 Gdansk, Poland; 2 Department of Pharmaceutical Biochemistry, Medical University of Gdansk, Debinki 1, 80-211 Gdansk, Poland; 3 Faculty of Chemistry, University of Gdansk, Wita Stwosza 63, 80-308 Gdansk, Poland; 4 Department of General, Endocrine and Transplant Surgery, Medical University of Gdansk, Smoluchowskiego 17, 80-214 Gdansk, Poland

## Abstract

Routine laboratory lipid assays include simple measurements of total cholesterol, triacylglycerols and HDL. However, lipids are a large group of compounds involved in many metabolic pathways, and their alterations may have serious health consequences. In this study, we used ^1^H NMR to analyze lipids extracted from sera of 16 obese patients prior to and after bariatric surgeries. We observed a post-surgery decrease in serum concentrations of lipids from various groups. The hereby presented findings imply that ^1^H NMR is suitable for rapid, simple and non-invasive detection of lipids from 30 structural groups, among them triacylglycerols, phosphatidylcholine, phosphatidylethanolamine, sphingomyelin, total phospholipids, total, free and esterified cholesterol, total and unsaturated fatty acids. NMR-based analysis of serum lipids may contribute to a substantial increase in the number of routinely determined markers from this group; therefore, it may find application in clinical assessment of obese subjects prior to and after bariatric surgeries, as well as in the examination of patients with other metabolic diseases.

## Introduction

Since the discovery of nuclear magnetic resonance (NMR) phenomenon and publications of E.M. Purcell *et al*.^1^ and F. Bloch *et al*.^2^ from 1946, due to progress in multi-dimensional, multi-quantum Fourier spectroscopy and magnetic resonance tomography, NMR has found application in many scientific disciplines^3^. Nowadays, NMR spectroscopy is widely used in clinical trials and everyday practice as a diagnostic tool to detect various biomarkers, especially those related to metabolic disorders^4–7^. Moreover, this technique is suitable for structural examination and rapid qualitative and quantitative analyses of non-derivatized lipids extracted from membranes, tissues and biofluids^5,8–10^. An advantage of NMR is its high reproducibility, which makes this method suitable for identification and validation of biomarkers^11^. Another important feature of NMR spectroscopy is a possibility to analyze the sample without its destruction; as a result, the sample can be used for repeated analyses and/or for sequential analyses of various analyte species^12^. However, according to some authors, NMR-based analyses should be accompanied by examination with MS techniques to improve the accuracy of results obtained in complex biological systems^11–13^.

**Figure 1:**
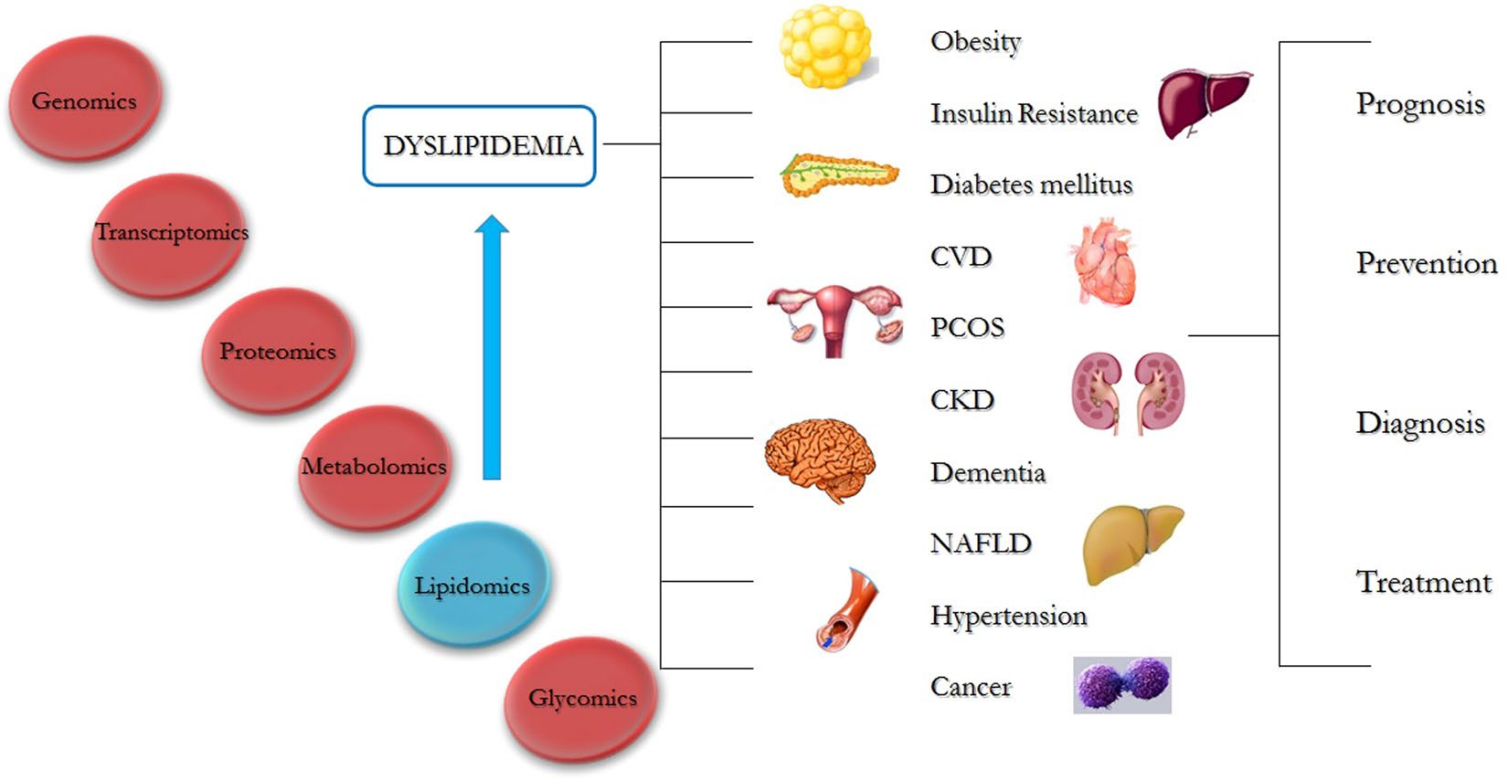
Use of lipidomics in the management of various diseases.

Lipidomics is a research discipline analyzing whole lipidome with high throughput and on a large scale^14^. In lipidomic studies, analytical technique is adjusted for matrix type, and according to some authors, NMR spectroscopy is the most promising method used for biofluid analysis^12,15^. The results obtained by lipidomic analyses may be applied to prognosis, prevention, diagnosis and treatment of various metabolic diseases (Fig. 1). Only few lipid biochemical markers, triacylglycerols (TGs), total cholesterol and HDL cholesterol, are assayed in routine practice, whereas other, more diverse groups of lipids, such as various classes of polar lipids, are not determined at all. In this study, we determined the levels of phospholipids (PL), including phosphatidylcholine (PC), phosphatidylethanolamine (PE) and sphingomyelin (SM), based on their characteristic head groups. Polar lipids, among them phospholipids, are crucial for structural integrity and interactions between molecules^16^. However, little is known about PL composition in obese patients, and the role of this lipid group in pathogenesis of metabolic diseases is still not fully understood^17^. Changes in polar lipid profile are observed during the course of many metabolic diseases, including obesity, and may be reflected by altered properties of plasma membranes^17^. Previous studies documented some changes in serum lipidome of obese patients, such as alterations of fatty acid composition^18^ and fatty acid content in phospholipids^17^. Furthermore, changes in SM and ceramide levels contribute to obesity-induced endothelial dysfunction and cardiovascular disease^19,20^. SM and ceramides are also involved in control of cell proliferation and/or cancer prevention^21^. Consequently, knowledge of specific lipid groups present in the serum is not only vital for the identification of related lipid disorders, but may also help to understand their impact on various metabolic processes.

Obesity is a major public health problem. In 2014, more than 600 million subjects worldwide were classified as obese, and more than 1.9 billion adults were overweight^22^. Epidemiological data suggest that in 2030, obesity may be present in 50% of general population^23^. This justifies research on novel treatment options, and puts emphasis on prevention of side effects of currently available therapies. Complex morbid obesity can be managed by dietary treatment, pharmacotherapy and bariatric surgery. Other than the two former strategies, bariatric surgery is highly effective, resulting in a significant reduction of food intake and/or decrease in digestion and absorption^24^. Furthermore, bariatric treatment was shown to significantly reduce cardiovascular risk and even more importantly, may contribute to remission of type 2 diabetes mellitus^25^. However, as any other therapy, also bariatric surgery may produce some adverse effects, such as peripheral neuropathy, metabolic bone disease, micronutrient deficiency and protein-calorie malnutrition^26,27^. Therefore, the aim of this study was to analyze the impact of bariatric surgery on the levels of lipids from selected groups being vital for health of obese patients. To achieve this goal, we used a new approach, NMR spectroscopy, as this method offers many advantages which are not available in the case of conventional laboratory techniques.

To this date, polar lipid contents in obese patients were determined with several techniques. Complex lipids, such as PC, PS, PE, PI and SM, were separated with EDTA vacutainers^28^, HPLC^29^, TLC^30,31^ and SPE^32^, whereas blood composition of FA in PHL was determined by means of GC^28,30–32^, LC-MS/MS^33^ and UPLC-MS^34^. All these techniques are suitable for detection, identification and quantification of lipids from several groups, but the analysis is time-consuming and includes multiple stages^35^. Moreover, we still lack an accurate method for rapid determination of whole spectrum of blood lipids. ^1^H NMR may be helpful in early detection of disorders in various groups of serum lipids being associated with a plethora of diseases, among them obesity. The main objective of this study was to develop a simple and rapid method capable to determine as many groups of serum lipids as possible. The study included patients with morbid obesity, examined prior to and after bariatric surgery; however, our hereby presented diagnostic method is likely also applicable to other metabolic diseases. We showed that extraction with chloroform and methanol mixture, followed by NMR analysis lasting no longer than a few minutes, is a time-sparing method that can detect up to 10 various groups of lipids, including TG, PC, PE, SM, total PL, total fatty acids and unsaturated fatty acids (UFA), as well as total, free and esterified cholesterol. Moreover, we were able to detect some specific lipid compounds, such as 7-lathosterol, oleic acid and linoleic acid.

## Results

### ^1^H NMR analysis of sera from obese patients prior to and after bariatric surgery

**Figure 2:**
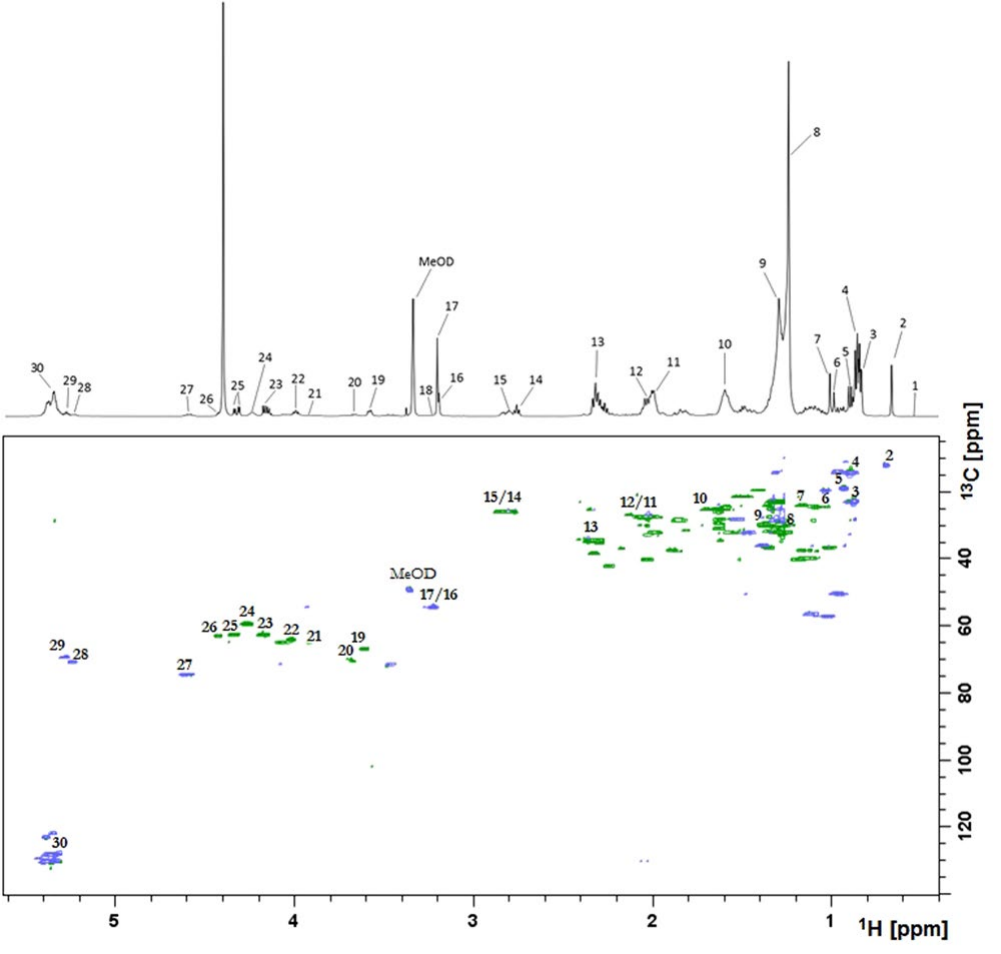
Upper panel: A representative ^1^H NMR spectrum of serum from obese patient, with resonance signals assigned to respective groups of lipids; bottom panel: HSQC spectrum. All detected structural groups are listed in Table 1.

The analysis included 16 sera samples from obese patients who underwent bariatric surgery. A representative ^1^H NMR spectrum of patient’s serum is shown on Fig. 2 (upper panel). A total of 30 structural groups of serum lipid extracts were identified and quantified (Table 1). A set of 2D NMR experiments (COSY, TOCSY, HSQC, HSQC-TOCSY, and HMBC) was recorded to confirm the chemical shifts assignment in ^1^H NMR spectra. The spectrum HSQC (Fig. 2 – bottom panel, and Supplementary Figure 1) showed linkages (through one bond) between protons and corresponding carbon atoms. The identification of signals in a complex ^1^H NMR spectrum using a combination of ^1^H and^13^C chemical shifts obtained from HSQC experiments is much more reliable. Moreover the section of TOCSY spectrum (Supplementary Figure 2) allowed to distinguish protons of TG and PL (two different spin systems - red and blue lines).

### Verification of ^1^H NMR analysis accuracy against routine laboratory lipid assays

**Figure 3:**
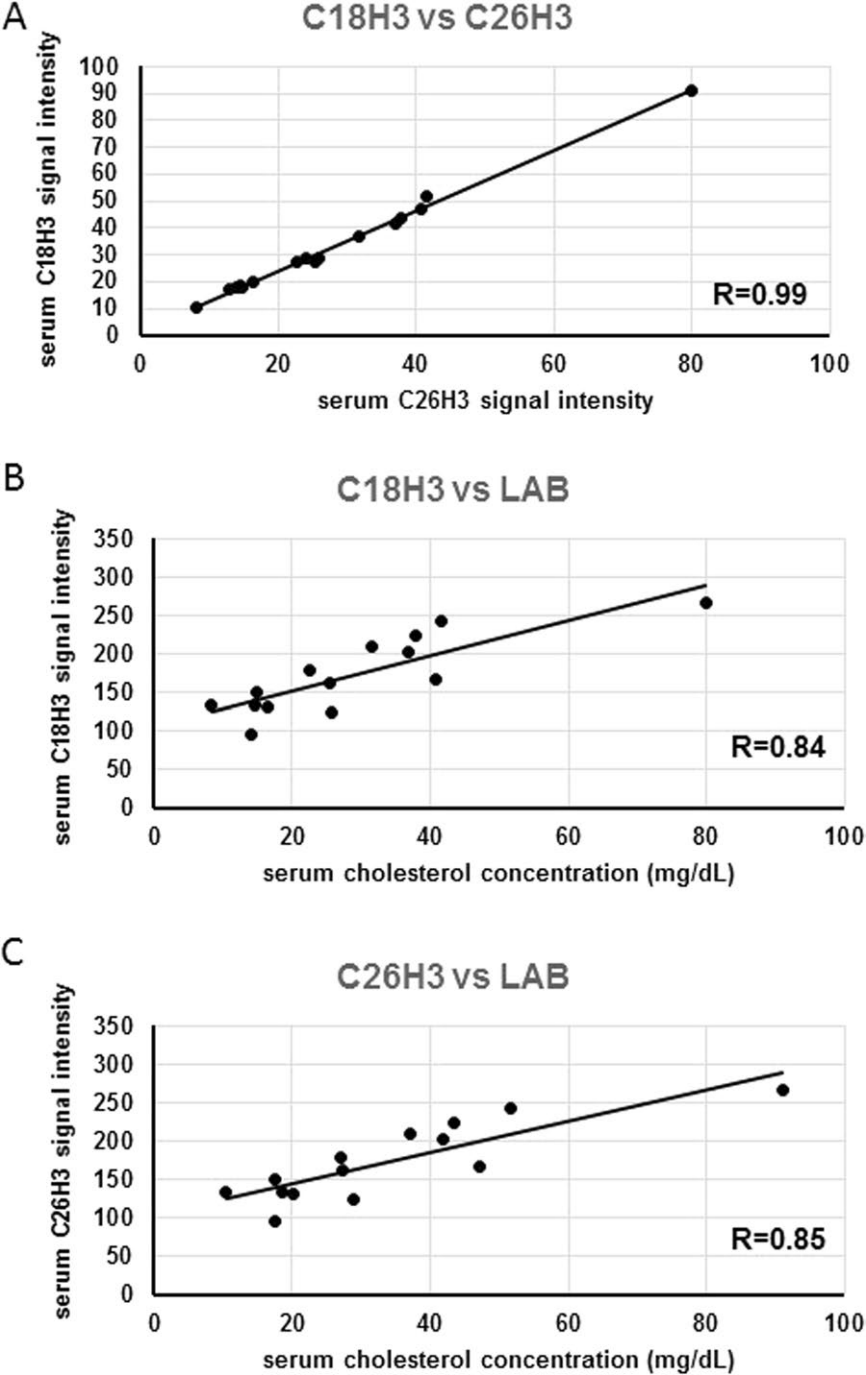
Associations between C_18_H_3_ and C_26_H_3_^1^H NMR signals for total cholesterol (**A**), C_18_H_3_^1^H NMR signal for total cholesterol and serum cholesterol concentration determined routinely at a clinical laboratory (**B**), C_26_H_3_^1^H NMR signal for total cholesterol and serum cholesterol concentration determined routinely at a clinical laboratory (**C**) in the study subjects.

**Figure 4:**
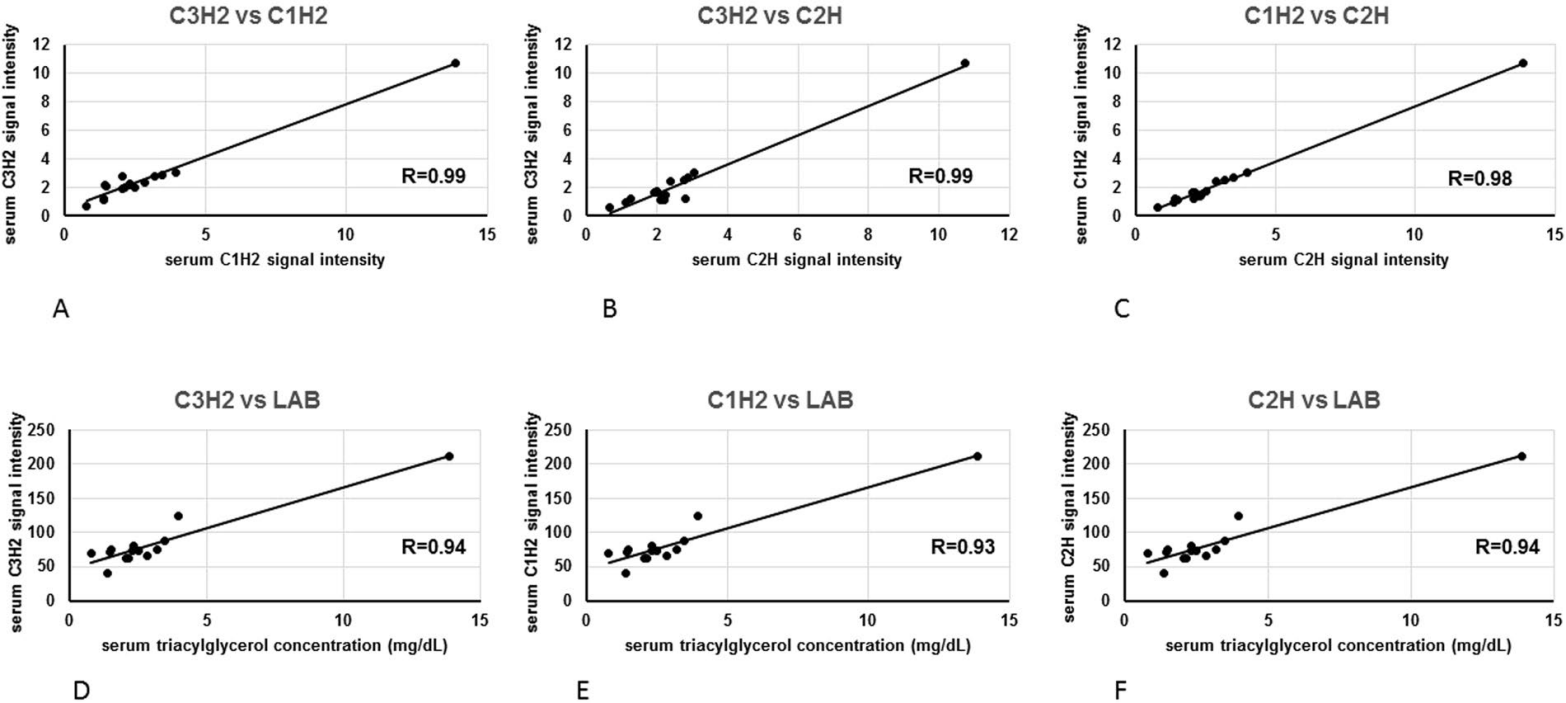
Associations between C_3_H_2_ and C_1_H_2_^1^H NMR signals for triacylglycerol (**A**), C_3_H_2_ and C_1_H_2_^1^H NMR signals for triacylglycerol (**B**), C_1_H_2_ and C_2_H^1^H NMR signals for triacylglycerol (**C**), C_3_H_2_^1^H NMR signal for triacylglycerol and serum triacylglycerol concentration determined routinely at a clinical laboratory (**D**), C_1_H_2_^1^H NMR signal for triacylglycerol and serum triacylglycerol concentration determined routinely at a clinical laboratory (**E**), C_2_H^1^H NMR signal for triacylglycerol and serum triacylglycerol concentration determined routinely at a clinical laboratory (**F**) in the study subjects.

Two groups of lipids, total cholesterol and triacylglycerols, were determined both by means of NMR and using routine laboratory methods. To verify the accuracy of NMR analysis, we analyzed correlations between the results obtained with both methods. As shown on Fig. 3, two NMR signals for total cholesterol correlated strongly with each other, and showed a strong positive correlation with serum concentration of cholesterol determined using routine laboratory method. Similarly, three NMR signals specific for triacylglycerols correlated strongly with one another, as well as with serum concentration of triacylglycerols determined routinely at our laboratory (Fig. 4).

### Changes in serum lipids after bariatric surgery

As shown in Table 1, bariatric surgery contributed to a significant decrease in the levels of all analyzed lipids, by 57% for triacylglycerols, by 40–45% for SM, PC, PE and by 42% for total PL. The levels of total, free and esterified cholesterol decreased by 43–48%, and the levels of total fatty acids and specific fatty acids, including UFA, 18:1, 18:2, 20:4 and 22:6, by about 50%. The most evident decrease (by about 80%) was observed in the case of C_18_H_3_ group in total 7-lathosterol (Table 1).

## Discussion

### ^1^H NMR as a simple method for rapid detection of changes in many groups of serum lipids in morbidly obese patients after bariatric surgery

**Figure 5:**
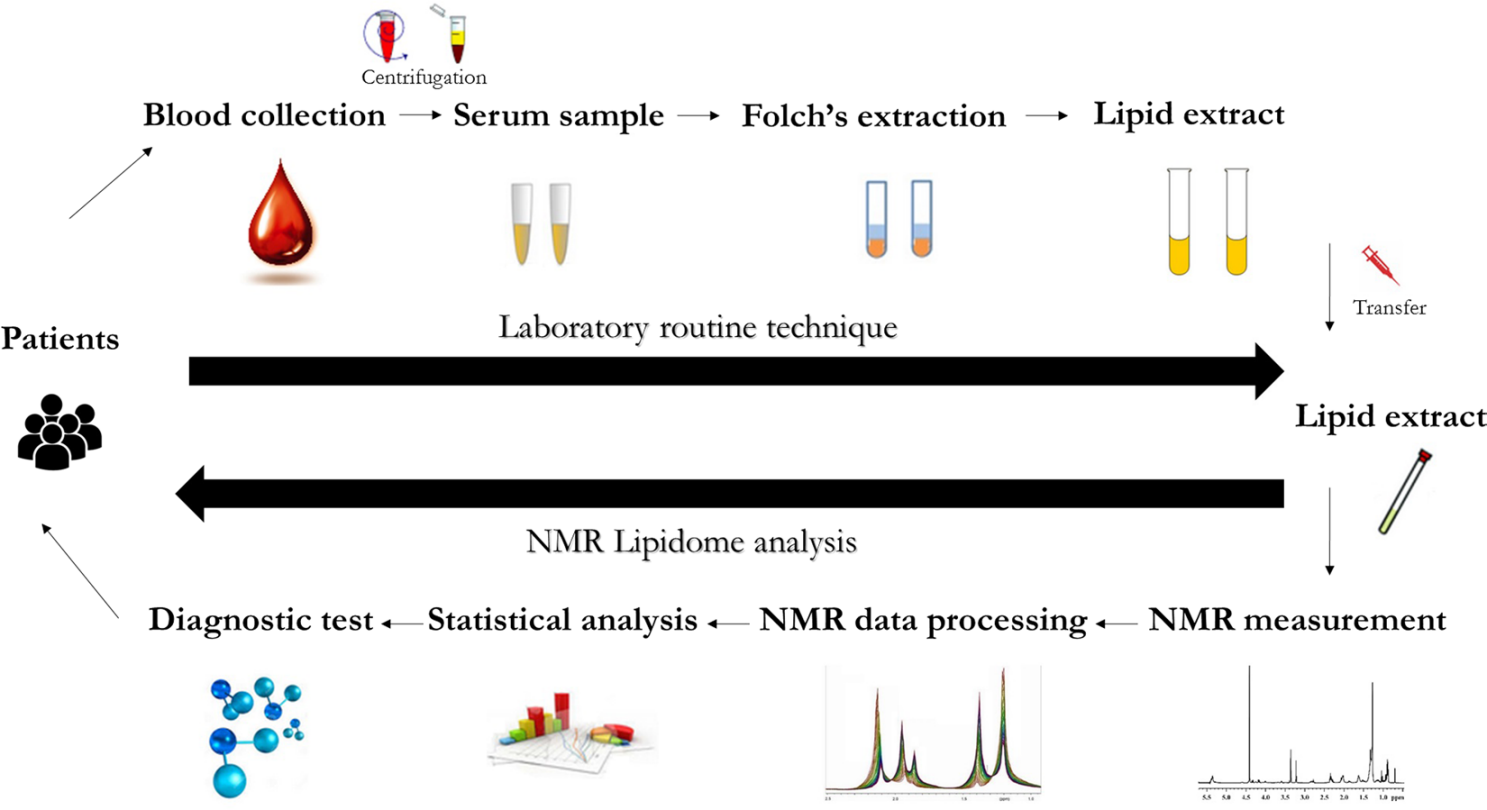
A conceptual image presenting the overview of the methodology used in this study. The most important advantage of this approach is significantly increased number of analyzed serum lipid species comparing to standard serum lipid assay in clinical laboratory. The measurement of these lipid species (e.g. phospholipids) is not possible by standard enzymatic assays. Moreover their measurement by our NMR method can be performed in just a few minutes.

Although NMR has been previously applied to the analysis of lipid contents in adipose tissue^36,37^ and blood serum^38^, this technique is relatively infrequently used in lipidology, mainly as a method to determine lipoprotein levels^16,39,40^. Only few previous studies used NMR for metabolomic analysis of sera from obese subjects prior to and after bariatric surgery^41–44^. In these studies, no lipid extraction was made and NMR analysis was conducted with intact blood sera, which resulted in identification of only few or none lipid species. In our present study, we first extracted serum lipids with Folch’ method^45^, and then analyzed the lipid extracts by means of NMR. This enabled us to detect 30 various structural groups of serum lipids. High accuracy of our method was confirmed by strong positive correlations between NMR signal intensity for 2 groups representing total cholesterol/3 groups representing triacylglycerols and serum concentrations of these metabolites determined using routine enzymatic methods. Postoperative changes in cholesterol and triacylglycerol contents were more evident when estimated on the basis of NMR spectra (see: Tables 1 and 2). This implies that our NMR analysis is more suitable to estimate the direction and magnitude of postoperative changes in various lipid species, than to determine their absolute concentrations. According to some authors, due to its low sensitivity and problems with obtaining satisfactory quality of a multi-parallel analysis of complex lipid extracts with hundreds of metabolites, NMR should not be used without a supporting MS, especially in the case of low-abundance analytes^46^. However, our present study showed that despite these potential limitations, NMR is suitable for rapid lipidomic analysis of whole groups of polar lipids: PC, PE, SM and total PL. Moreover, this technique may provide an information about a global direction of changes in lipidome of patients after bariatric surgeries. Obtaining such information by means of MS would be much more costly and time-consuming. All this makes NMR lipid analysis more promising from a clinical perspective. Another NMR approach was employed by researchers from Computational Medicine Research Team from Finland, who has developed a high-throughput serum NMR metabolomics platform, which by combining NMR analysis of three molecular windows: LMWM (low- molecular-weight metabolites), LIPO (lipoproteins) and LIPID (extracted lipids) is able to measure total of 233 variables, including serum lipids^47,48^. This platform has been employed for metabolomics analyses in many clinical studies conducted in patients with early atherosclerosis, coronary heart disease, metabolic syndrome, type 1 and 2 diabetes mellitus and diabetic nephropathy^47^. It would be certainly worth doing such metabolomics analyses in morbidly obese patients before and after bariatric surgery. In turn, our method is quite simple, but also allows for rapid identification of the most important groups of serum lipids. Moreover it allows to measure some additional serum lipids – PE as well as lathosterol, which can serve as a marker of cholesterol production rate in human organism^49^. In Fig. 5 we present the overview of the methodology used in this study.

**Table 1:** Mean changes (Λ) in lipid classes level 6 months after bariatric surgery.

Resonance No	^1^H NMR signal	Chemical shift (ppm)	Λ (%)	Mean Λ (%) of common signals of lipids
**1**	–C18**H3** in total 7-lathosterol	0.55	−82.5	
**2**	–C18**H3** in total cholesterol	0.70	−44.2	TOTAL CHOLESTEROL −48.4
**3**	–C26**H3/**-C27**H3** in total cholesterol	0.86	−52.7	
**5**	–C21**H3** in free cholesterol	0.93	−43.9	FREE CHOLESTEROL −44.2
**6**	–C19**H3** in free cholesterol	1,02	−44.7	
**7**	–C19**H3** in esterified cholesterol	1.04	−44.1	ESTERIFIED CHOLESTEROL −43.2
**27**	–3C**H** in esterified cholesterol	4.60	−42.4	
**4**	–C**H3** in fatty acyl chain	0.89	−51.8	TOTAL FATTY ACIDS −49.4
**8**	–(C**H2**)n in fatty acyl chain	1.27	−50.4	
**10**	–CO-CH2C**H2-** in fatty acyl chain	1.61	−47.0	
**13**	–CO-C**H2-** in fatty acyl chain	2.3	−48.3	
**9**	=CHC**H2**CH2(CH2)- in fatty acyl chain	1.32	−49.6	UNSATURATED FATTY ACIDS −49.7
**30**	–**HC**=**CH-** in fatty acyl chain	5.37	−49.8	
**11**	–C**H2**HC= in fatty acyl chain: 18:1	2.03	−48.6	
**12**	–C**H2**HC= in fatty acyl chain: 18:2/20:4	2.08	−48.8	
**14**	–CH**CH2**CH= in fatty acyl chain: 18:2	2.78	−47.5	
**15**	–CH**CH2**CH= in fatty acyl chain:20:4/22:6	2.84	−53.3	
**18**	–C**H2**–CH2–NH2 of PE	3.26	−44.8	PHOSPHATIDYLETHANOLAMINE −42.1
**21**	–>C2**H** in glycerol backbone of PE	3.92	−39.4	
**17**	–N+ (C**H3**)3 in PC head group	3.22	−43.8	PHOSPHATIDYLCHOLINE −41.1
**20**	–C**H2**N + (CH3)3 in PC head group	3.68	−38.4	
**22**	>C3**H2** in glycerol backbone of PL	4.01	−39.9	PHOSPHOLIPIDS −41.6
**26**	–C1**H2** in glycerol backbone of PL	4.43	−39.9	
**28**	–C2**H** in glycerol backbone of PL	5.24	−44.9	
**25**	>C1**H2**/C3**H2** in glycerol backbone of TG	4.33	−56.2	TRIACYLGLYCEROLS −57.2
**29**	–>C2**H** in glycerol backbone of TG	5.28	−58.3	
**16**	–N + (C**H3**)3 in SM head group	3.21	−47.2	SPHINGOMYELINS −44.7
**19**	–C**H2**N + (CH3)3 in SM head group	3.62	−43.3	
**24**	–C**H2**CH2N + (CH3)3 in SM head group	4.25	−43.6	
**23**	>C1**H2/**C3**H2** in glycerol backbone of TG and PL	4.16	−51.5

**Table 2:** Biochemical and anthropometric characteristics of the study subjects.

	Before BS (Mean ± SEM)	6 months after BS (Mean ± SEM)
Age (years)	44 ± 3.1	—
BM (kg)	119 ± 4.6	91 ± 3.6*
BMI (kg/m^2^)	41 ± 1.1	31 ± 1.1*
Triglycerides (mg/dL)	119 ± 22	83 ± 11*
Total cholesterol (mmol/L)	190 ± 11	179 ± 12*
HDL (mmol/L)	45 ± 2.6	52 ± 2.8*
LDL (mmol/L)	122 ± 9.5	110 ± 10*
Creatinine (mg/dL)	0.84 ± 0.04	0.73 ± 0.03*
Insulin (µU/mL)	17 ± 3.7	5.6 ± 0.47*
Glucose (mg/dL)	98 ± 11	86 ± 2.1
Albumin (g/L)	40 ± 0.81	39 ± 0.53

BM – Body mass

BS - Bariatric surgery

*p < 0.05.

### Bariatric surgery contributes to a decrease in serum lipid content

Our study demonstrated that in morbidly obese patients, bariatric surgery contributed to a significant decrease in all analyzed groups of serum lipids. However, the degree of the reduction was group-specific. The most evident decrease (by 82.5%) was observed in the case of 7-lathosterol, a precursor of cholesterol. 7-lathosterol is a marker of cholesterol production rate^49^, and therefore, our findings suggest that bariatric surgery results in a substantial decrease in cholesterol synthesis. This observation is consistent with the results published recently by Vuono *et al*.^50^, who using GC-MS, also demonstrated a significant decrease in serum lathosterol level in patients after sleeve gastrectomy. The same study revealed that lower level of serum lathosterol was associated with a decrease in cholesterol synthesis. After bariatric surgery, the levels of total, esterified and free cholesterol in our patients were approximately 45% lower than prior to the procedure. However, even more pronounced (57%) postoperative reduction was observed in the case of triacylglycerol content. Noticeably, also the analysis with routine enzymatic methods demonstrated that the postoperative decrease in TG was greater than the reduction of total cholesterol level (Table 2). The key deliverable of our study is the development of a method for rapid detection of quantitative changes in many groups of polar lipids, including PC, PE, SM and total PL, as well as UFA in sera from morbidly obese patients after bariatric surgeries. UFA are the major component of PL^51^. The intensities of UFA and PL signals after bariatric surgery were shown to correlate strongly (R = 0.95; p < 0.01), which implies that the postoperative decrease in PL might result from a lesser availability of UFA. Morbid obesity is associated with enhanced oxidative stress^52^, and UFA are particularly prone to peroxidation^51^. Products of lipid peroxidation play a role in the pathogenesis of insulin resistance, diabetes mellitus^53^, chronic inflammation, atherosclerosis, cardiovascular diseases^51^ and some disorders incorporated into metabolic syndrome^54^; all these conditions may be also associated with morbid obesity. Therefore, the postoperative decrease in UFA and PL may contribute to attenuation of oxidative stress, a phenomenon observed after bariatric surgeries^55^. Aside from the role in oxidative stress propagation, PL composition and content may also influence the risk of diabetes mellitus^29^. Therefore, a method for routine determination of their serum levels in obese subjects would have unquestioned clinical value. Although polar lipids seem to be vital for health of obese subjects^17^, only few previous studies addressed bariatric surgery-related changes in serum phospholipids. Kayser *et al*.^56^ used HPLC-MS/MS to document a decrease in various species of PC, PE and PG at 3 months after gastric bypass procedure. Also in that study, a decrease in the level of SFA-containing species of SM co-existed with an increase in the content of PUFA-containing SM^56^. A decrease in PC, PE and SM was also reported by Graessler *et al*.^57^ three months after RYGB procedure. Both studies mentioned above were based on MS techniques and therefore, provided an information about the contents of specific species from each group of polar lipids, which was not possible in the case of our method. However, in contrast to the MS-based technique, our method is suitable for rapid, non-destructive analysis of a sample; thus, it provides an information about the changes in overall PC, PE and SM contents in the sample, which can be then re-used for more accurate MS analyses, if necessary. Contrary to our hereby presented findings, Lopes *et al*.^42,43^ reported an increase in PC at 12 months after RYGB. Perhaps, PC content increases with time elapsed since the bariatric procedure, due to a raise in HDL concentration. Our study demonstrated a ca. 50% postoperative decrease in the levels of total and unsaturated fatty acids, as well as a decrease in the contents of two specific fatty acids, oleic and linoleic. This is not surprising, owing that fatty acids are the main component of other, more complex lipids. Interestingly, Forbes *et al*.^32^ reported an increase in serum concentration of FFA at one month after bariatric surgery, which might correspond to enhanced postoperative catabolism of lipids; however, FFA have eventually returned to their baseline level at 6 months post-procedure. FFA constitute only a small proportion of all FA building other serum lipids, including PL and TG. Oleic acid is synthetized primarily in the liver, by stearoyl-CoA desaturase (SCD1)^58^. Therefore, a postoperative decrease in the concentration of this FA might reflect lower rate of liver lipogenesis and FA desaturation after bariatric surgery. On the other hand, linoleic acid is an essential fatty acid and thus, its decreased content after bariatric surgery was likely associated with a reduced food intake.

In conclusion, this study showed that extraction of serum lipids, followed by NMR analysis, lasting no longer than a few minutes, are suitable for rapid detection of various serum lipids, including total TG, PC, PE, SM, total PL, total, free and esterified cholesterol, total and unsaturated fatty acids, as well as 7-lathosterol, oleic acid, and linoleic acid, in obese patients prior to and after bariatric surgeries. A postoperative decrease in serum levels of various lipids likely reflected their reduced dietary intake, as well as lower rate of cholesterol production, and less intense liver synthesis and desaturation of fatty acids in obese patients subjected to bariatric surgeries. However, a concomitant decrease of some other lipid species being important for patient health, such as PUFA and PL, points to a necessity of their dietary supplementation in patients after bariatric surgery, in order to prevent deficiencies. NMR-based analysis of serum lipids may contribute to a substantial increase in the number of routinely determined markers from this group; therefore, it may find application in clinical assessment of obese subjects prior to and after bariatric surgeries.

## Material and Methods

### Patients

Sixteen obese patients (7 men and 9 women with mean BMI of 41 ± 1.1 kg/m^2^) aged 27–61 years (mean age, 44 ± 3.1 years) underwent bariatric surgery (BS) at the Department of General, Endocrine and Transplant Surgery, Medical University of Gdansk (Poland). Six patients were subjected to sleeve gastrectomy (SG), another 6 to Roux-en-Y gastric bypass (RYGB), and 4 to omega-loop gastric bypass (OLGB). Patients included in the study didn’t present clinical evidence of endocrine, cardiac, hepatic or renal diseases, additionally all of them were non-smokers. Anthropometric and laboratory parameters of the study subjects were determined twice, prior to the surgery and 6 months thereafter. Blood samples for NMR analysis were obtained after an overnight fast. Standard laboratory parameters were determined at the Central Clinical Laboratory, Medical University of Gdansk. The study conformed to the principles of the Declaration of Helsinki of the World Medical Association. Protocol of the study was approved by the Local Bioethics Committee at the Medical University of Gdansk (decision no. NKEBN/281/2014), and prior to the enrolment, written informed consent was sought from all the study subjects. All tests used in this study followed relevant protocols and guidelines. Selected biochemical and anthropometric characteristics of the study subjects are presented in Table 2.

### Sample preparation

Total lipids were extracted from whole serum samples with a chloroform-methanol mixture (2:1, v/v), as described previously^59^. The extraction process lasted 30 minutes. Prior to lipidomic analysis, serum analytes were dissolved in 600 µl of deuterated chloroform and deuterated methanol mixture (2:1; v/v) with 3 mM TMS. After centrifugation, the samples were transferred to 5-mm NMR tubes, and stored at 4 °C until the analysis.

### NMR measurement and data processing

All NMR spectra were recorded with Bruker Avance III 500 MHz spectrometer at 298 K and referenced to TMS signal (0.00 ppm). The ^1^H NMR spectra of lipid extracts from patient sera were recorded using *zg30* pulse sequence. Each spectrum was comprised of 64 consecutive scans, with acquisition time of 4 s, FID size of 65 k and spectral width of 15 ppm.

2D NMR homonuclear ^1^H, ^1^H COSY (correlation spectroscopy) and TOCSY (total correlation spectroscopy), as well as 2D NMR heteronuclear ^1^H,^13^C HSQC (heteronuclear single quantum coherence), HSQC-TOCSY (heteronuclear single quantum coherence - total correlation spectroscopy) and HMBC (heteronuclear multiple bond correlation) experiments were recorded to confirm the chemical shifts assignment.

The obtained ^1^H NMR spectra were processed with 0.3 Hz line broadening, manually phased and corrected for baseline distortion with MestReNova software (Mestrelab Research v. 11.0). The essential signals in ^1^H NMR spectra were integrated manually. All obtained integrals were scaled according to the area of the TMS reference signal.

### Statistical analysis

Statistical significance of differences in pre- and postoperative values of the study variables was verified with paired t-test. The differences were considered significant at p < 0.05. Relationships between pairs of variables were determined on linear regression analysis. All statistical calculations were carried out with Sigma Stat software.

## Electronic supplementary material

Supplementary information
