# Supplementary material for: Potential Application of 1H NMR for Routine Serum Lipidome Analysis –Evaluation of Effects of Bariatric Surgery
Source: Sci Rep. 2017 Nov 14;7:15530. doi: 10.1038/s41598-017-15346-0 (PMC5686116; doi:10.1038/s41598-017-15346-0)
Supplement: Supplementary file 1 — Supplementary information [file 41598_2017_15346_MOESM1_ESM.pdf]

Supplementary material

Potential Application of ^{1}H NMR for Routine Serum Lipidome Analysis – Evaluation of Effects of Bariatric Surgery

Adriana Mika[1,2,*], Zbigniew Kaczynski[3], Piotr Stepnowski[1], Maciej Kaczor[4], Monika Proczko-Stepaniak[4], Lukasz Kaska[4], Tomasz Sledzinski[2]

[1] Department of Environmental Analysis, Faculty of Chemistry, University of Gdansk, Wita Stwosza 63, 80-308 Gdansk, Poland

[2] Department of Pharmaceutical Biochemistry, Medical University of Gdansk, Debinki 1, 80-211 Gdansk, Poland

[3] Faculty of Chemistry, University of Gdansk, Wita Stwosza 63, 80-308 Gdansk, Poland

[4] Department of General, Endocrine and Transplant Surgery, Medical University of Gdansk, Smoluchowskiego 17, 80-214 Gdansk, Poland

Corresponding author:

* Dr. Adriana Mika; Department of Environmental Analysis, Faculty of Chemistry, University of Gdansk, Wita Stwosza 63, Gdansk, 80-308; Poland; tel./fax: +48585230810,

Email: adrianamika@tlen.pl

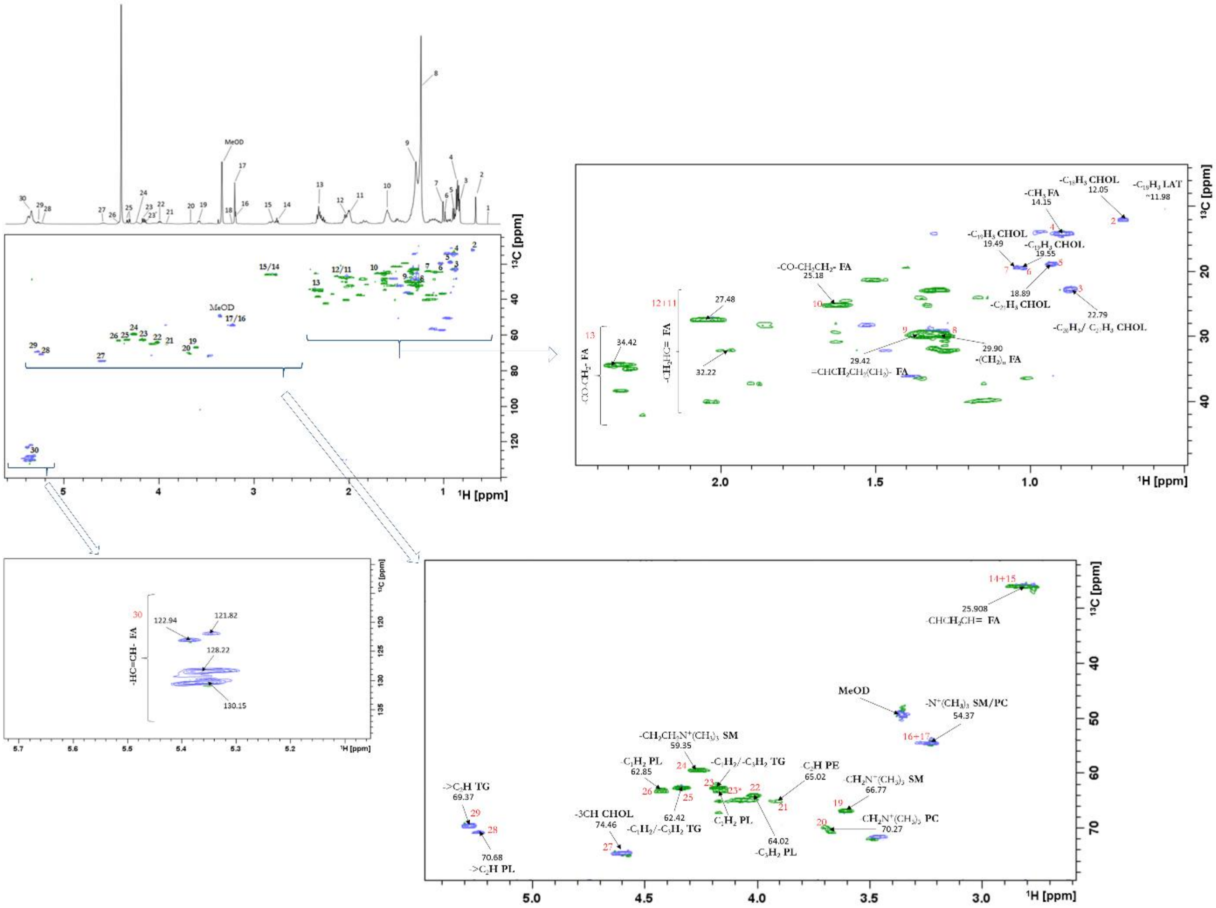

Figure S1. The typical ^{1}H-^{13}C HSQC spectrum of MO patients' serum with described ^{13}C chemical shifts. Signal 23 * comes from >C1H2 PL and is common part of signal 23. It was also included at Supplementary Figure S2 online, which presented particular position of PL and TG protons (D' proton).

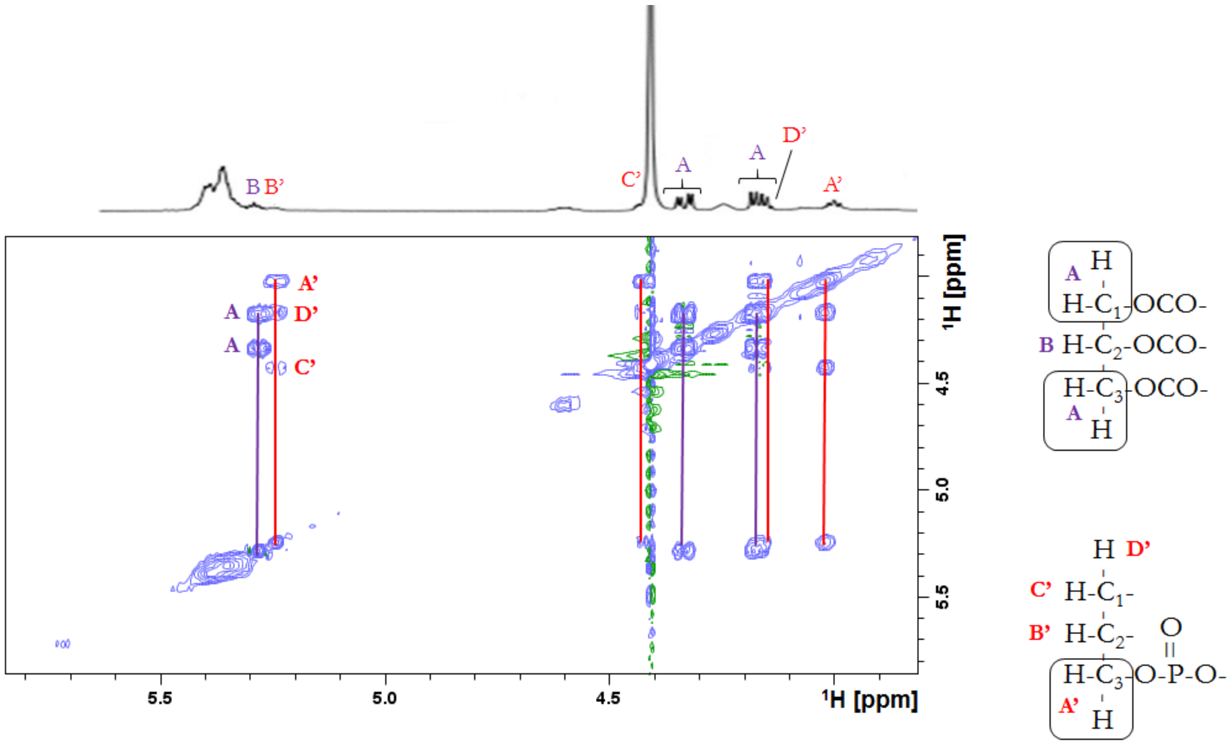

Figure S2. The ^{1}H-^{1}H TOCSY spectrum of MO patients' serum determining the position of TG and PL protons. TG - violet lines; PL - red lines.
